# Lysis‐lysogeny coexistence: prophage integration during lytic development

**DOI:** 10.1002/mbo3.395

**Published:** 2016-08-17

**Authors:** Qiuyan Shao, Jimmy T. Trinh, Colby S. McIntosh, Brita Christenson, Gábor Balázsi, Lanying Zeng

**Affiliations:** ^1^ Department of Biochemistry and BiophysicsTexas A&M UniversityCollege StationTexasUSA; ^2^ Center for Phage TechnologyTexas A&M UniversityCollege StationTexasUSA; ^3^ Department of Biology and BiochemistryUniversity of Northwestern ‐ St. PaulSt. PaulMinnesotaUSA; ^4^ Laufer Center for Physical & Quantitative BiologyStony Brook UniversityStony BrookNew YorkUSA; ^5^ Department of Biomedical EngineeringStony Brook UniversityStony BrookNew YorkUSA

**Keywords:** bacteriophage lambda, cellular decision‐making, DNA labeling, *Escherichia coli*, lysis‐lysogeny, prophage integration

## Abstract

The infection of *Escherichia coli* cells by bacteriophage lambda results in bifurcated means of propagation, where the phage decides between the lytic and lysogenic pathways. Although traditionally thought to be mutually exclusive, increasing evidence suggests that this lysis‐lysogeny decision is more complex than once believed, but exploring its intricacies requires an improved resolution of study. Here, with a newly developed fluorescent reporter system labeling single phage and *E. coli* DNAs, these two distinct pathways can be visualized by following the DNA movements *in vivo*. Surprisingly, we frequently observed an interesting “lyso‐lysis” phenomenon in lytic cells, where phage integrates its DNA into the host, a characteristic event of the lysogenic pathway, followed by cell lysis. Furthermore, the frequency of lyso‐lysis increases with the number of infecting phages, and specifically, with CII activity. Moreover, in lytic cells, the integration site *attB* on the *E. coli* genome migrates toward the polar region over time, leading to more spatial overlap with the phage DNA and frequent colocalization/collision of *attB* and phage DNA, possibly contributing to a higher chance for DNA integration.

## 1 Introduction

Cellular decision‐making is a ubiquitous process among all organisms, from the most complicated metazoans to the simplest biological systems such as viruses, with bacteriophage lambda being one of best‐studied model systems. Upon infection by bacteriophage lambda, *E. coli* cells can enter one of two distinct pathways, lysis or lysogeny; this decision‐making process,celebrated as the “genetic switch” (Ptashne, 2004), has been extensively studied at the population level (Wuff & Rosenberg 1983, Court et al., 2007; Dodd et al., 2005; Oppenheim et al., 2005). The lytic pathway leads to immediate and rapid phage propagation with cell death and release of hundreds of progeny, while the lysogenic pathway features continued cell growth and passive replication of phage DNA after its integration into the host chromosome. Historically, this “lysis versus lysogeny” decision has been considered as mutually exclusive, where lysogeny is favored in nutrient‐poor environments, as low quantity and quality of host cells results in suboptimal phage propagation (Kourilsky, 1973). Therefore, the lysogenic pathway provides an alternative mechanism for the virus to store its DNA until favorable environments for propagation arise in the future. The lysis‐lysogenic decision‐making represents an evolutionary strategy of diversification for the virus, allowing it to react to and thrive in variable conditions, to maximize its own fitness.

The protein players involved in this cellular decision‐making process have been well‐characterized over decades (Court et al., 2007; Dodd et al., 2005; Oppenheim et al., 2005), and CII, Cro and and Q are among the key proteins that determine the infection outcome, mediating either the lysogenic or lytic pathways (Oppenheim et al., 2005). Cro facilitates the lytic pathway by being a weak repressor for phage gene expression from both pL and pR promoters (Folkmanis et al., 1977; Kobiler et al., 2005; Svenningsen et al., 2005; Takeda et al., 1977), while Q activates the lytic pathway after reaching a threshold, allowing for the expression of a single transcript carrying the lysis and morphogenesis genes (Kobiler et al., 2005; Marr et al., 2001). Conversely, CII activation will inhibit the lytic pathway and establish the lysogenic pathway by activating transcription from three specific promoters (Kobiler et al., 2005; Oppenheim et al., 2005). Among them, the pI promoter allows the expression of the lambda integrase, Int, which catalyzes the crucial process of integrating phage DNA into the host chromosome (Landy, 1989; Nash, 1981).

New details have emerged from higher‐resolution studies of this well‐established system (St‐Pierre & Endy, 2008; Van Valen et al., 2012; Zeng et al., 2010). Our recent study performed at the single‐cell level proposed that individual phages infecting the same cell are able to “vote” for the cell's fate independently (Zeng et al., 2010), which raised the possibilities that lytic and lysogenic pathways can happen simultaneously within the same cell, resulting from the different votes by multiple infecting phages. This coexisting lytic‐lysogenic development may be naturally beneficial, serving as an intermediate state allowing for a faster and more sensitive commitment to lysis‐lysogeny in a changing environment. Exploring this phenomenon requires a higher resolution of study and can yield insights into the biological process of decision‐making and its evolutionary strategy.

In this study, we developed an improved reporter system at the single‐DNA level to allow the visualization of phage DNA integration, in addition to the progress of the lytic and lysogenic pathways. By tracking phage and host DNA movements after infection in real‐time using fluorescence microscopy, and quantitatively analyzing single‐molecule trajectories, we reveal a new biological phenomen on of “lyso‐lysis” and gain further insights into the possible mechanism of cellular decision‐making.

## 2 Experimental Procedures

### 2.1 Bacterial strains

Bacterial strain LZ722 was constructed by inserting a DNA array containing ~200 *tetO* repeats into strain LZ220 (Shao et al., 2015) at ~1,500 bp upstream of *attB* site using lambda red recombination (Datsenko & Wanner, 2000). Plasmid pFtsKi‐*tetR‐mCherry*, which contains the *tetR‐mCherry* under the constitutive promoter FtsKi was transformed into LZ722, resulting in LZ731. For all real‐time microscopy experiments, LZ731 is used as the host, while for bulk assays (lysogenization, PCR and qPCR), *E. coli* strain MG1655 is used. Unless otherwise specified, phage titration assays for determining the phage concentration was done with *E. coli* strain LE392.

### 2.2 Plasmid construction

To construct the plasmid pLZ729: pFtsKi‐*tetR‐mCherry*, plasmid pWX510 (Wang et al., 2014) was digested with HindIII and BamHI restriction enzymes to obtain sequences for *tetR‐mCherry*, which was then inserted into pBR322. DNA sequences for FtsKi was PCR amplified from pWX6 (Wang et al., 2005) using primers QS15 and QS16 and inserted into the above plasmid between EcoRI and HindIII recognition sites, resulting in EcoRI‐FtsKi‐HindIII‐*tetR‐mCherry*‐BamHI in the pBR322 backbone. When this plasmid was transformed into LZ722, the background signal (mCherry) was found to be too high, therefore we switched to another vector, pACYC177, which has a lower copy number. The piece FtsKi‐HindIII‐*tetR‐mCherry* was PCR amplified using primers QS17 and QS18 and inserted into pACYC177 between SmaI and NheI, resulting pLZ729. The plasmid pZA32‐*dam* carries the *dam* gene in between AvrII and KpnI in the pZA32 backbone, where the *dam* gene was amplified with primers QS19 and QS20, using template plasmid pGG503 (Herman & Modrich, 1981). When phages were produced from *dam* ^*+*^ host cells containing this plasmid, pZA32‐*dam*, the phage DNA was confirmed to be fully methylated (Fig. S1D).

### 2.3 Phage strains

The phage λ *D‐mTurquoise2 cI*_*857*_ *bor::Kan* ^*R*^ was obtained through recombination by infecting λ *Dam cI*_*857*_ *bor::Kan* ^*R*^ on the permissive strain LE392‐bearing plasmids pBR322‐*D‐mTurquoise2‐E*. The recombinant (λ *D‐mTurquoise2 cI*_*857*_ *bor::Kan* ^*R*^) was selected based on its ability to titer on nonpermissive strain MG1655 and fluoresce under a fluorescence dissecting microscope. For easier selecting and counting of lysogens for λ*int* ^*−*^ in the lysogenization assays, λ*int* ^*−*^ *‐Kan* was constructed following the protocol as described in (Shao et al., 2015) to replace the nonessential *bor* gene region of λ*int* ^*−*^ with a *Kan* ^*R*^ cassette.

### 2.4 Phage lysate preparation

Fully methylated mosaic phage λWT‐FP was obtained by inducing a lysogen with temperature‐sensitive prophage (λ *D‐mTurquoise2 cI*_*857*_ *bor::Kan* ^*R*^) and two plasmids, plasmid pPLate‐*D* to provide wild‐type phage decorative capsid protein gpD (Zeng et al., 2010) and plasmid pZA32‐*dam* which over produces Dam methylase after 1 mmol/L IPTG induction. Fully methylated phages λWT, λ*int* ^*−*^, λ*cII*_*68*_ , and λ*cII*_*stable*_ were obtained by infecting host cell LE392 carrying plasmid pZA32‐*dam* with the corresponding phages at 42°C. This is important if the phage lysate will be used for quantifying the lyso‐lysis using qPCR. We found that the phage lysate obtained through prophage induction contains nonnegligible amount of integrated phage DNA, possibly due to insufficient induction, while the phage lysate obtained through infecting the host cells contains no integrated DNA. All phage lysates used were also purified following the protocols described in (Zeng & Golding, 2011).

### 2.5 Bulk lysogenization assay

To measure the lysogenization frequency of the various phages, we followed the protocol as described in (Zeng et al., 2010). For easier selection and counting of lysogens, the phage λ*int* ^*−*^ *‐Kan* was used instead of λ*int* ^*−*^ since *bor* gene was reported to be nonessential and would not affect the lysogenization frequency (Barondess & Beckwith, 1995). All the other phages used also carried an antibiotic marker by replacing the λ *bor* region. Briefly, 2 ml of the host cell MG1655 was grown in LBMM for overnight and subsequently diluted 1:1,000 into 12 ml of LBMM and grown to OD_600_ ~ 0.4 at 37°C, centrifuged (2,000*g* for 10 min at 4°C) and resuspended to be ~1.5 × 10^9^ cells ml^−1^ in prechilled LBMM (LB + 0.2% maltose + 10 mmol/L MgSO_4_). Thereafter, 20 μl of the resuspended cells were then infected with 20 μl of phages at different concentrations by incubation for 30 min on ice. The samples were then transferred to 35°C water bath for 5 min to allow for phage DNA ejection, followed by 10‐fold dilution into prewarmed LBGM (LB + 0.2% glucose + 10 mmol/L MgSO_4_) and incubation with shaking at 265 rpm at 30°C for 45 min. The samples were then properly diluted and plated on LB plates containing appropriate antibiotics to allow ~100 colonies on each plate.

### 2.6 PCR and qPCR

Here 2 ml of host cell MG1655 was grown in LBMM overnight and was subsequently diluted 1:1,000 into 100 ml of LBMM and grown to OD_600_ ~ 0.4 at 37°C. Cells were then spun down at 2,000*g* for 10 min at 4°C and resuspended to be ~1.5 × 10^9^ cells ml^−1^ in prechilled LBMM. Infection was set up following the same protocol described in Bulk lysogenization assay, with corresponding phages at different concentrations for infections of different APIs, but with larger volumes depending on the number of samples to be taken later (100 μl of reaction per sample). For each time point, a quantity of 100 μl of the reaction was added to 0.9 ml prewarmed LBGM shaking at 265 rpm in 30°C shaker for various times up to 120 min. For confirming and quantifying lyso‐lysis, samples were taken at each time point and immediately filtered using 0.2 μm membrane to obtain cell‐free samples. For the infection with different APIs, the samples taken at 90 min were used, and samples were diluted 10‐fold into dH_2_O to minimize possible PCR inhibitor effects. PCR or qPCR was performed immediately after the last sample was taken. PCR was done using primers in (Powell et al., 1994), while qPCR was done using primers QS1 and QS2 for detecting *E*. *coli* DNA, and primers QS3 and QS4 for detecting integration (Table S2). Amplification was done using SYBR Green PCR master mix (Applied Biosystems, 4309155) with 250 nmol/L of each primer. For determining the mRNA level of *int/xis/cII*, infection was done following the same protocol, but with 5× volumes for each sample. Samples were taken out at different time points: 0, 6, 12, 18, 24, 30, and 40 min, and immediately poured into 5 ml ice‐cold methanol. Samples were then spun down at 4,000*g* for 4 min, at 4°C. The cell pellet was resuspended in 1 ml of RNAprotect Bacteria Reagent (Qiagen, 76506), followed by incubation for 5 min at room temperature. Then the cells were spun down at 5,000*g* for 5 min at room temperature. After discarding the supernatant, the cells were kept at −20°C until all samples were collected. RNA extraction was done using RNeasy Mini Kit (Qiagen, 74104), followed by DNA digestion with TURBO DNA‐free kit (ambion, AM1907) for a total of 80 min and reverse transcription using High Capacity RNA‐to‐cDNA kit (Applied Biosystems, 4387406). The obtained cDNA was then quantified, using SYBR Green PCR master mix. Primers QS7 and QS8 were used for quantifying *cII*, primers QS9 and QS10 for *int* and primers QS11 and QS12 for *xis*, while *ihfB* is used as a reference gene using primers QS5 and QS6 (Table S2).

### 2.7 Quantifying percentage of multiple prophage integration

Infection was set up as described in Bulk lysogenization assay, with the infecting phages being λWT at API of 0.1, 1 and 10. After obtaining the lysogens on the plates, 96 colonies of each infection were used to determine whether they contain single or multiple phage integration by PCR following protocols as described in (Powell et al., 1994). The percentage of cells having multiple prophage integration is then calculated based on the PCR results.

### 2.8 Microscopy

A quantity of 1 ml of host cell LZ731 was grown in M9 minimal medium (11.3 g l^−1^ M9 salts, 1 mmol/L MgSO_4_, 0.5 μg ml^−1^ thiamine HCl, 0.1% casamino acids, 100 μmol/L CaCl_2_) supplemented with 0.4% maltose (M9M) with appropriate antibiotics for overnight. Here 60 μl of the culture was subsequently diluted 1:100 into 6 ml M9M and grown to OD_600_ ~ 0.4. 1 ml of cells were then collected by centrifugation at 2,000*g* for 2 min at room temperature, and resuspended in 40 μl of M9M. Thereafter, 20 μl of phage lysate was then added to 20 μl of cells to reach an API of 0.5–5, followed by incubation for 30 min on ice and another 5 min at 35°C water bath to allow DNA ejection. The sample was then diluted into M9M at room temperature by 10‐fold. 1 μl of the diluted sample was used for imaging following protocols as described in (Shao et al., 2015) with 1.5% M9M agarose pad. Imaging was performed on an inverted microscope (Ti‐E, Nikon, Tokyo, Japan) with a cage incubator (InVivo Scientific, St. Louis, MO) set at 30°C. Images were taken using 100× objective (Plan Fluo, NA 1.40, oil immersion) with standard filter sets and a cooled EMCCD camera (iXon 3 897, Andor, Belfast, United Kingdom). When needed, a series of 9 z‐stack images with spacing of 300 nm in the CFP channel (200 ms exposure) was taken to capture all infecting phages in the initial frames, after which images were taken every 5 min through the phase‐contrast, YFP, mCherry, and CFP channels (100, 200, 50 and 100 ms exposure respectively) at the focal plane to allow tracking of DNA movement and cell fate in the time‐lapse movies.

### 2.9 Data analysis

Images were processed using MicrobeTracker (Sliusarenko et al., 2011). Briefly, cells were first outlined using MicrobeTracker, after which spots were recognized first automatically using SpotFinderZ, then manually corrected using SpotFinderM (Sliusarenko et al., 2011). Cell lineage tracking and the calculation of minimum distance between *attB* and lambda DNA, Dis(λ‐*attB*), for each cell was done using custom Matlab script in our lab. The Dis(λ‐*attB*) is calculated as the minimum distance between all possible pairs of lambda DNA and *attB* in each given cell at each given time point, where the distance between lambda DNA and *attB* was calculated as: $\sqrt{{\left(x_{i}-m_{j}\right)}^{2}+{\left(y_{i}-n_{j}\right)}^{2}},$ where *i*,* j* = 1, 2, 3 up to the total number of lambda DNA or *attB* in each cell at each time point, and *x*_*i*_, *y*_*i*_ are the *x* and *y* coordinates of lambda DNA, while *m*_*j*_ and *n*_*j*_ and are those of *E. coli attB*.

## 3 Results

### 3.1 Reporter system for phage DNA integration: *E. coli* attB and phage DNA labeling

**Figure 1:**
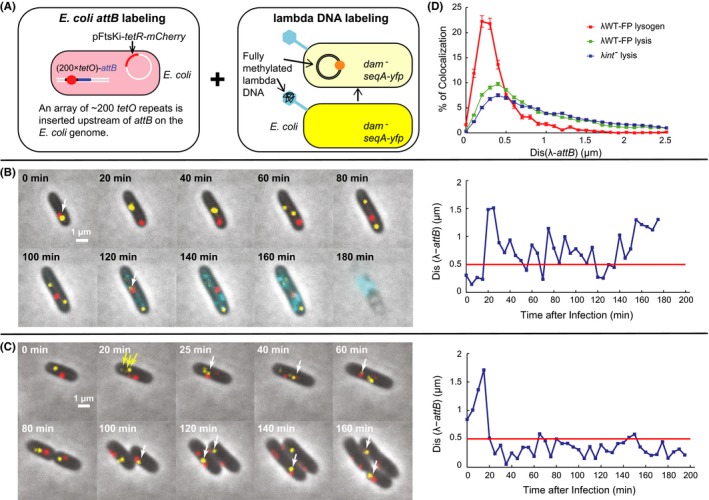
Lambda DNA and *E. coli attB* fluorescent reporters allow DNA tracking in lytic and lysogenic cells. (A) Schematic diagram describing the reporter system. Left, the *E. coli attB* appears as a red dot reported by about 200 *tetO* repeats upstream of *attB* bound by TetR‐mCherry expressed from plasmid pFtsKi‐*tetR*‐mCherry. Right, the DNA of a gpD‐mTurquoise2 (cyan) labeled phage appears as a yellow dot when ejected into a *dam* ^*−*^ *seqA‐yfp* cell. (B and C). Overlay images of representative lytic (B) and lysogenic (C) events respectively, with the corresponding right panel showing the minimum distance between *attB* and lambda DNA foci, Dis(λ‐*attB*), over time (blue line) with a 0.5 μm cutoff line (red). White arrows point to the colocalized lambda DNA and *attB*. (B) The lambda DNA and *attB* do not colocalize most of the time in this lytic cell, although occasionally, that is, at 0 and 120 min from the selective images, colocalization apparently occurs, possibly due to random collision or imaging artifact. (C) Yellow arrows point to lambda DNA observed at 20 min. DNA colocalization (white arrows) was observed starting from 25 min in this lysogenic cell. (D) Distribution of Dis(λ*‐attB*) for lytic and lysogenic cells after λWT‐FP infection and lytic cells after λ*int* ^*−*^ infection. The lysogenic cells have a higher peak at shorter distances (0–0.5 μm) compared to the lytic cells, while λ*int* ^*−*^ infected lytic cells show a flatter distribution. Error bars represent ± SEM.

In the lysogenic pathway, lambda DNA is integrated into the *E. coli* genome at the *attB* site through recombination by the phage‐encoded integrase, Int, in the presence of the host factor IHF (Nash, 1981). To visualize this integration event, we developed a reporter system to simultaneously track the *E. coli attB* and the phage DNA. Specifically, the host cell LZ731 contains about 200 repeats of *tetO* (Lau et al., 2003) inserted upstream of *attB* on the chromosome (Fig. 1A, left) and a plasmid pFtsKi‐*tetR‐mCherry*, which constitutively expresses TetR‐mCherry (Wang et al., 2005), therefore the *tetO* repeats are bound by TetR‐mCherry, resulting in a distinct spot (Fig. 1A–C, red dots), indicating the *attB* location. The phage DNA is labeled using our previously reported method (Shao et al., 2015) (also see Fig. 1A, right). Briefly, the phage λWT‐FP was produced in a host with enhanced Dam methylase activity resulting in fully methylated phage DNA packaged in its head (see Experimental Procedures). The host cell LZ731 also constitutively expresses a fluorescent SeqA chimera, SeqA‐YFP (Babic et al., 2008) from the chromosome, and the host DNA is not methylated owing to a *dam* ^*−*^ mutation (methylation deficient). SeqA specifically binds to fully methylated and hemi‐methylated DNA, so the phage DNA appears as a YFP spot (Fig. 1A–C, yellow dots) once ejected into the cell. The phage DNA reporter system allows tracking of the first two copies of each initial DNA after replication, since there are only two methylated strands of DNA. For example, in Figure 1B, the yellow focus splits into two at 60 min, and no new foci appear despite continued DNA replication. Cells with more than two foci, e.g. three foci in Figure 1C at 20 min, indicated by yellow arrows, are presumably infected by more than one phage. The phage λWT‐FP (here referred to as WT for simplicity and easier comparison with the *int* and *cII* mutants used later, and FP is used to indicate this phage is labeled with fluorescent proteins. See detailed genotype in Table S2) also carries a *D‐mTurquoise2* marker, which encodes a chimera of the gpD decorative capsid protein fused to the mTurquoise2 fluorescent protein (Goedhart et al., 2012); this enables the monitoring of the lytic development by imaging cyan fluorescence (Zeng et al., 2010), as observed in Figure 1B.

With this reporter system, the location and movement of the lambda DNA and *attB* can be tracked over time. Cells entering the lytic and lysogenic pathways are expected to show no (or very short‐term) colocalization and long‐term colocalization respectively. In Figure 1B, the cell entered the lytic pathway, indicated by accumulation of gpD‐mTurquoise2 (120–180 min) and cell lysis (180 min). This lytic cell occasionally showed short‐term colocalization at 0 and 120 min (see also Movie S1), which could be due to random collision or just imaging artifact. In contrast, long‐term colocalization was observed for cells entering the lysogenic pathway. For example, in Figure 1C (see also Movie S2), one pair of phage DNA and *attB* colocalized beginning at 25 min, and another pair at 40 min, showing long‐term colocalization. This cell later divided and cell growth continued, indicating that the cell entered the lysogenic pathway. Occasional apparent separation of phage DNA and *attB* after long‐term colocalization was also observed for lysogenic cells, for example, at 80 min in Figure 1C. The lambda DNA is ~48 kbp in length, and the SeqA 5′‐GATC‐3′‐binding sites are relatively evenly distributed across the lambda genome (Fig. S1A). Therefore, due to the uncertainty of the sites bound by SeqA‐YFP on the lambda DNA and the movement of the bound unit resulting from diffusion (Weber et al., 2010), coupled with the fact that the *tetO* repeats are located ~1,500 bp upstream of *attB*, the actual distance between mCherry/*attB* and YFP/lambda DNA focus is expected to vary even after integration. This is probably why the *attB* and lambda DNAs are sometimes seemingly separated while the integration appears to have already happened.

To quantitatively determine colocalization, we then calculated the minimum distance between lambda DNA and *attB*, or Dis(λ‐*attB*) at each time point for each cell. For the lytic and lysogenic examples in Figure 1B and C, Dis(λ‐*attB*) of the lytic cell was usually above 0.5 μm (Fig. 1B), whereas in the lysogenic cells (Fig. 1C), it generally remained below 0.5 μm after integration (here beginning at 25 min). Moreover, the distribution of Dis(λ‐*attB*) across all time points during the time‐lapse movies for all lysogenic (*N* = 44) and lytic cells (*N* = 515) showed that the lysogenic cells exhibited Dis(λ‐*attB*) in the range of 0–0.5 μm much more often than lytic cells (Fig. 1D), suggesting that 0.5 μm might be a good indicator for determining lambda/*attB* colocalization. In fact, for all lysogenic cells, after the designated integration time, we found that Dis(λ‐*attB*) largely stayed below 0.5 μm over the remaining time course of the movie (Fig. S1B); therefore we defined “spot colocalization” as having a Dis(λ‐*attB*) below 0.5 μm.

### 3.2 Lyso‐lysis: cell lysis with phage DNA integration

**Figure 2:**
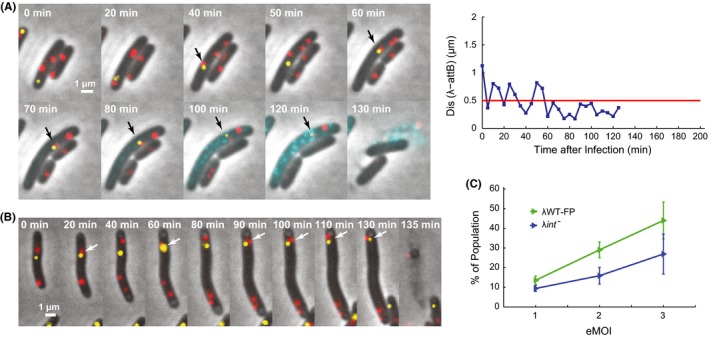
Apparent DNA integration is observed in some lytic cells. (A) After the λWT‐FP infection, a lytic cell shows a DNA integration event. DNA colocalization is observed starting from 60 min until cell lysis indicating DNA integration in lytic cells, which we name as lyso‐lysis. Black arrows indicate colocalized lambda DNA and *E. coli attB* site. The right panel shows the Dis(λ‐*attB*) along time. (B) Overlay images of a cell infected by λ*int* ^*‐*^ mutant show DNA colocalization before lysis. DNA colocalization occurs at 40 and 60 min, followed by separations right after. Starting from 90 min, lambda DNA and *attB* stay colocalized until the cell lyses at 135 min, leading to a false lyso‐lysis event. White arrows indicate colocalized lambda DNA and *E. coli attB* site. (C) The percentage of lyso‐lysis increases with eMOI for both λWT‐FP and λ*int* ^*−*^, with λWT‐FP showing a much higher percentage than λ*int* ^*−*^. Error bars represent ± SEM.

Interestingly and surprisingly, we observed some cells entering the lytic pathway while also showing long‐term colocalization of lambda DNA and *attB*. An example is shown in Figure 2A (see also Movie S3), where DNA colocalization occurred from 60 min until cell lysis (130 min), suggesting that phage DNA integration might be happening. Although unexpected, this event is actually consistent with the unanimous voting model proposed recently (Zeng et al., 2010), which states that each infecting phage in a cell can make a decision toward lytic or lysogenic independently. We then termed this event as “lyso‐lysis”.

Before quantifying DNA integration in lytic cells, we first excluded the contribution of random collision between lambda DNA and *E. coli attB* particles to the observed “colocalization”. Here we used phage mutant λ*int* ^*−*^ as a reference/control. λ*int* ^*−*^ has a mutation in the integrase, which makes it defective in integration and lysogenization (Fig. S2). As expected, the Dis(λ‐*attB*) distribution for λ*int* ^*−*^ (*N* = 510) showed significantly lower frequencies at 0–0.5 μm (Fig. 1D) compared to both the λWT‐FP lysogenic and lytic cells. This integrase‐dependent activity suggested that the observed DNA colocalization are likely due to the real DNA integration with some background of random collision. To our surprise, we noticed that λ*int* ^*−*^ infection sometimes also led to apparent lyso‐lysis events. For example, in Figure 2B (see also Movie S4), DNA colocalization happened at 90 min and lasted until cell lysis at 135 min after λ*int* ^*−*^ infection. We then compared the quantitative difference between colocalization for λWT‐FP and λ*int* ^*−*^ infected cells. A relaxed criterion was then set up to call out cells with apparent “integration”, for both λWT‐FP and λ*int* ^*−*^ infections. As long as the Dis(λ‐*attB*) is below 0.5 μm, in the last 15 min before lysis, the cell would be categorized as lyso‐lysis. At the same time, the effective number of phages infecting the cell (or effective Multiplicity of Infection, or eMOI) can be obtained by counting the initial phage DNA number. We then obtained the frequency of lyso‐lysis (calculated as number of lyso‐lytic cells over total cells) at each eMOI. As expected, phage λ*int* ^*−*^ infections led to lower percentages of lyso‐lysis at all eMOIs compared to λWT‐FP, although still showing a non‐negligible number of apparent lyso‐lysis events (Fig. 2C). Nevertheless, it suggests that lyso‐lysis does exist in λWT‐FP infections, although the frequency of lyso‐lysis may be overestimated due to the contribution of false colocalization events reported by the system and allowed by our criterion.

### 3.3 *E. coli* attB migrates toward the cell pole in lytic cells, leading to more colocalization with lambda DNA

**Figure 3:**
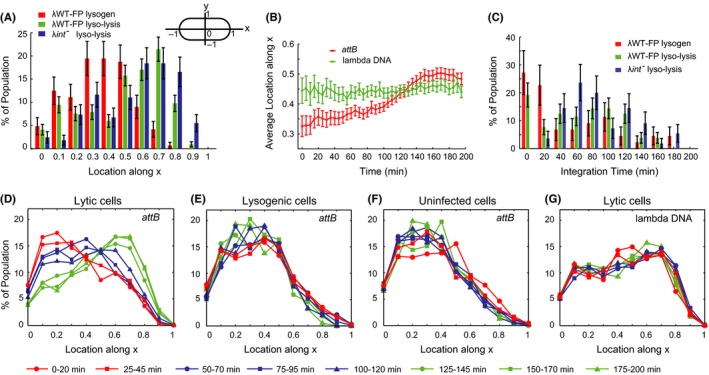
*E. coli attB* migrates to the polar region in lytic cells where lambda DNA preferentially locates. (A) The distribution of locations for colocalization. The diagram on the top right corner specifies the coordinates of cells used. The absolute value for the location along x/y is shown in all the panels. For lyso‐lytic cells, data were collected from the last 15 min before lysis, while for lysogenic cells, data were from the last 15 min of the movie (185–200 min). It shows that for lytic cells colocalization happens more often toward the cell pole, while in lysogenic cells it shows preference to the mid‐quarter cell region. (B) Average *attB* and lambda DNA locations along time after infection for λWT‐FP lytic cells. Lambda DNA location stays relatively unchanged at around quarter‐cell region, while the location of *attB* shifts gradually from mid‐quarter region toward the lambda DNA. (C) The distribution of integration times for lysogenic and lyso‐lytic cells. The integration for λWT‐FP lysogens happens mostly within the first 20 min with an average of 56 min. λWT‐FP lyso‐lytic cells integrate at an average of 68 min while the negative control, λ*int* ^*−*^ takes 89 min on average. (D, E and F). show the distribution of *attB* in lytic (D), lysogenic (E) and uninfected cells (F) along time after infection by λWT‐FP. In lytic cells, *attB* migrates toward the cell pole while in lysogenic and uninfected cells, the *attB* distribution remains the same. (G) The distribution of lambda DNA along time for lytic cells after λWT‐FP infection. The lambda DNA prefers the mid‐polar cell region and the distribution stays the same throughout the whole lytic developmental process. Error bars represent ± SEM.

To determine the quantitative differences between phage/*E*. *coli* DNA colocalization in lysogenic cells with true integration events and those reported lyso‐lysis events for λWT‐FP and λ*int* ^*−*^ under our criterion, we analyzed the DNA trajectories of both lambda and *attB* over time. We noticed that lytic cells showed colocalization of *attB* and lambda DNA at the cell pole more often than at other positions, similar to that of lyso‐lysis by λ*int* ^*−*^ infection shown in Figure 2B, where both lambda DNA and *attB* migrated toward the cell pole with time and eventually colocalized near the pole. In fact, when comparing the position of colocalization between lytic cells, 15 min before lysis, and lysogenic cells, from 185 to 200 min after infection (when phage DNA has already integrated and spot‐tracking stops), we observed a significant difference (Fig. 3A). Colocalization happens most frequently between mid‐cell and quarter‐cell positions for lysogens, while in lyso‐lytic cells, the location shifts drastically toward the cell pole.

We then ask whether the spatial colocalization patterns of λWT‐FP‐infected lytic cells result from natural preferences in *attB* and lambda DNA location during lytic development. In fact, the *attB* location distribution for lytic, lysogenic, and uninfected cells showed that the *attB* position shifted gradually toward the poles in lytic cells over time (Fig. 3D), but not in lysogenic (Fig. 3E) or uninfected cells (Fig. 3F). Interestingly, the phage DNA preferentially locates at the quarter‐cell region, without significant changes along time in the lytic pathway (Fig. 3G), indicating that as the lytic cycle progresses, the *attB* moves gradually to a region where lambda DNA preferentially locates. Consistent with this hypothesis, when comparing the average lambda DNA and *attB* locations along time, it was obvious that *attB* migrated toward the lambda DNA and subsequently crossed lambda DNA traces (Fig. 3B). Therefore, the false lyso‐lysis that we detected from λ*int* ^*−*^ and some of λWT‐FP infection were likely due to *attB* and lambda DNA being in close proximity to one another, especially toward the end of the lytic cycle. If this is the case, we expect that the DNA colocalization in the false lyso‐lysis events would happen later compared to the actual integration events. We then compared the apparent “integration” times (when the colocalization started) for lyso‐lysis and lysogenic cells (Fig. 3C). Integration in lysogenic cells happened mostly within 20 min after infection under our experimental conditions, which agreed with previously reported data (Freifelder & Levine, 1973), although late integration was also observed, leading to an average integration time of 56 min. Nevertheless, it was clear that integration happened later for lyso‐lytic cells on average, with those from λ*int* ^*−*^ infection showing the most significant difference with an average integration time of 89 min, while λWT‐FP infection showing 68 min on average. In fact, very few lyso‐lysis events from λ*int* ^*−*^ infection showed early integration within the first 20 min, in contrast to λWT‐FP lysogenic and lyso‐lysis events, although the two phages shared similar lysis times (Fig. S1C). Taken together, these findings suggest that as *attB* migrated to the cell poles, it would occupy a similar cellular region as lambda DNA, especially at later infection times, leading to the false lyso‐lysis from λ*int* ^*−*^ infection and some of the λWT‐FP infections. The shift of *attB* distribution in the lytic development could simply be a result of combination of cellular division inhibition, lack of host DNA replication, and compromised length extension (see Discussion). The underlying mechanism remains to be investigated.

### 3.4 Lyso‐lysis: a process regulated by CII

**Figure 4:**
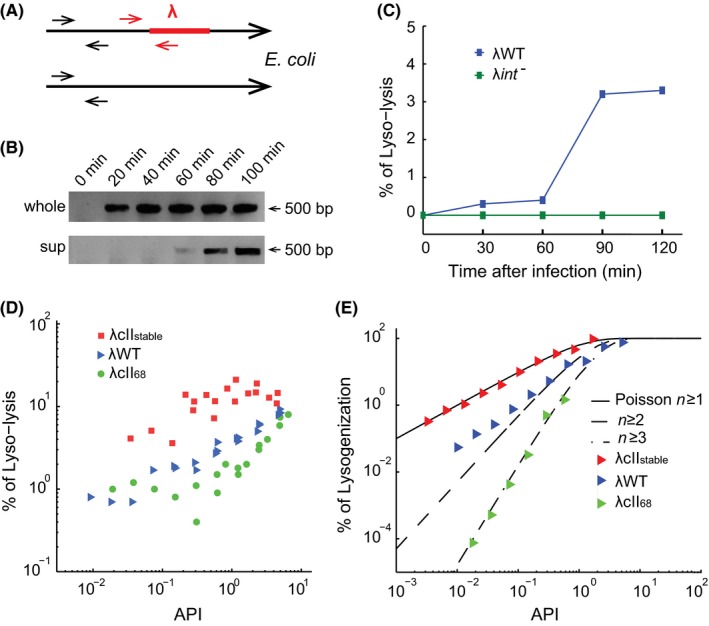
Probability of lyso‐lysis increases with API and CII activity. (A) A diagram showing the primer design for PCR and qPCR. For probing the integration using PCR, the primers span the junction between *E. coli* chromosome and lambda DNA, the integration junction, as indicated by red arrows, amplifying 500 bp in length. For qPCR, a different set of primers with the similar design is used. Another pair of primers is used for quantifying the *E. coli* DNA number, as indicated by black arrows. (B) PCR shows lyso‐lysis events. *E. coli* was infected by λWT with an API of 1, and samples were taken every 20 min after infection for PCR. PCR was done either using the sample directly (upper lane, labeled as “whole”) for detecting DNA integration from the whole sample, or using filtered supernatant to detect DNA integration in the lysed content (lower lane, “sup”). The 500 bp band indicating DNA integration shows up after 20 min in the “whole” sample as expected, and after 60 min in the “sup” sample indicating the lyso‐lysis events. (C) The lyso‐lysis frequency of λWT and λ*int* ^*−*^ along time by qPCR at an API of 1. λWT: blue, λ*int* ^*−*^: green. No amplification of DNA integration is detected for λ*int* ^*−*^ infection throughout the whole infection process (0–150 min). For λWT infection, the frequency of lyso‐lysis increases with time, with 60–90 min showing a drastic increase, corresponding to the time for cell lysis and releases of DNA for detection. (D) Lyso‐lysis is regulated by CII and has increased probability as API increases. Combined data of three qPCR experiments were shown. The frequency of lyso‐lysis for all three phages including λ*cII*_*6*8_, λWT and λ*cII*_*stable*_ increases with API and the effective CII level inside the cell. The frequency of lyso‐lysis follows the trend of λ*cII*_*68*_ **< λWT < λ*cII*_*stable*_, which is equivalent to their relative CII activities, suggesting that the process is regulated by CII. (E) The lysogenization frequency of λ*cII*_*68*_, λWT and λ*cII*_*stable*_ follows different trends. The data for each phage is shifted with respect to API to compare with the Poisson distribution. λ*cII*_*stable*_ (red right triangle) follows Poisson distribution of *n* ≥ 1 (black solid line), while λWT (blue right triangle) follows *n* ≥ 2 (black dashed line) and λ*cII*_*68*_ (green right triangle) follows *n* ≥ 3 (black dash dotted line), suggesting that the cell needs 1, 2, or 3 of the corresponding phages to lysogenize.

We designed a PCR experiment to examine whether *E. coli* genomic DNA liberated from lytic cells contains evidence of phage DNA integration as a complement to our microscopy data, as our reporter system does not specifically examine covalent DNA integration. We used primers specifically targeting the junction of *E. coli* and lambda DNA, spanning the *attL* region (Fig. 4A, red arrows) to confirm integration (Powell et al., 1994). Phage infection was done with an API (average phage input; the ratio of phages to cells) of 1, and samples were taken every 20 min after infection (see Experimental Procedures). Samples (containing all lytic, lysogenic and uninfected cells) were then either used directly (Fig. 4B, upper lane) as a positive control, or spun down and filtered to obtain the lysate (cell‐free, containing the medium and the cellular content of lysed cells) for PCR (Fig. 4B, bottom lane). As shown, DNA integration was first observed 20 min after infection (Fig. 4B, upper lane) in lysogenic and/or lyso‐lytic cells, while for the cell‐free lysate, integration was detected starting from 60 min after infection (Fig. 4B, bottom lane), corresponding to the time when cells began to lyse under these conditions, to release their DNA into the environment to be detected. Therefore, this suggested that DNA integration and thus lyso‐lysis happened in some lytic cells. We then further quantified the percentage of lyso‐lysis (defined as number of integrated DNA over total *E. coli* DNA) in the lytic cells, using qPCR with additional primers to quantify the *E. coli* DNA number (Fig. 4A, black arrows). Consistently, the percentage of lyso‐lysis increased significantly between 60 and 90 min to 3.5% at an API of 1 (Fig. 4C). λ*int* ^*−*^ was used as a negative control and no DNA integration was detected, as expected (Fig. 4C). These results further support the notion that phage DNA integration does occur in lytic cells. The number calculated here can be an underestimation since there may be multiple copies of *E. coli* DNA per cell at the time of infection, and not all copies will have phage DNA integration in lyso‐lytic cells.

We further explored the molecular mechanism underlying this lyso‐lysis phenomenon. CII promotes the expression of *int* from pI promoter in addition to activating the transcription of repressor CI from the pRE promoter for establishing the lysogenic pathway and inhibiting lytic gene expression through the anti‐sense transcript from paQ (Oppenheim et al., 2005). It is therefore possible that transient CII activation of pI without activation of pRE or paQ leads to the lyso‐lysis that we observe, without producing enough CI and antisense Q transcript to establish a stable lysogen. Lyso‐lysis may therefore be enhanced through common factors that lead to increased CII activity, such as higher APIs. Indeed, we found that as API increased, the percentage of lyso‐lysis increased for λWT infection (Fig. 4D), similar to that of lysogenization (Kourilsky, 1973; Zeng et al., 2010). Since the CII activity correlates with its protein level (Kobiler et al., 2005), more CII might lead to more Int expression and thus more lyso‐lysis. We then compared the percentage of lyso‐lysis of λWT to two phage mutants, λ*cII*_*68*_ and λ*cII*_*stable*_. λ*cII*_*68*_ carries a mutation which makes CII unable to dimerize to function (Datta et al., 2005; Schwarz et al., 1978), and λ*cII*_*stable*_ is less susceptible to FtsH degradation and is more stable (Jones & Herskowitz, 1978). Therefore the average CII activity after infection with the same API is: λ*cII*_*68*_ **< λWT < λ*cII*_*stable*_. As expected, the frequency of lyso‐lysis followed the same trend: λ*cII*_*68*_ **< λWT < λ*cII*_*stable*_ (Fig. 4D). Another interesting phenomenon was that the slope of lyso‐lysis frequency as a function API (at log‐log scale) is inversely related to the effective CII levels, showing the trend: λ*cII*_*68*_ **> λWT > λ*cII*_*stable*_, which is the same as that of the lysogenization frequency for these phages (Fig. 4E). Altogether, this suggests that similar to the lysogenic pathway, lyso‐lysis is also regulated by the CII activity.

## 4 Discussion

Cellular decision making is an important process for the fitness and survival of all organisms, and has recently attracted numerous studies (Balazsi et al., 2011). Temperate phages, one of the simplest biological systems display lysis‐lysogeny decision making after infecting the host bacterium. Classically, these decisions have been described as leading to incompatible outcomes, despite that early after infection, to proceed down one pathway without going at least part way down the other is impossible since early genes favoring lytic and lysogenic pathways are expressed from the same promoters, making the two pathways interconnected (Wulff & Rosenberg, 1983). In this work, by specifically labeling the lambda DNA and *E. coli attB* locus to allow for the direct visualization of prophage integration, we found that some lytic cells also have lambda DNA integration, previously thought to be present only in the lysogenic pathway. This finding offers a new perspective to the fundamental process of cellular decision making by bacteriophage lambda that phages cannot only concurrently develop two distinct pathways in the early infection period, but are also able to reach both outcomes in the late developmental stage.

Our recent model proposed that phages infecting the same *E. coli* cell can make individual votes to determine the cell's outcome, and unanimous voting by all infecting phages is a requirement for lysogeny (Zeng et al., 2010). This means that for those cells infected with more than one phage, it is possible that some phages vote lysogenic and the others lytic, leading to cell lysis, and observations of lyso‐lysis support this model. However, our data also suggest that lyso‐lysis exists even with one single phage infection (Figs. 2C, 4D), which is counterintuitive according to this unanimous voting model. It may be possible that phage voting occurs at the level of single phage DNA, where DNA replication early after infection provides additional deciding units, which can then decide different fates to result in lyso‐lysis. This scenario is in fact supported by our observation that 71% (235 out of 330 eMOI = 1 infections) of the very first two copies of replicated DNAs separate from each other and move to different locations inside the cell. Moreover, the key protein for lytic development, Q, has been reported to function largely *in cis* (Echols et al., 1976), suggesting that the localization of Q might be restricted. Therefore, if enough physical separation of the DNA happens early before a decision is reached, each DNA might maintain its individuality and be able to make a different decision since one phage DNA committing to the lytic pathway would not be expected to force other DNAs to vote lytic due to the restricted localization of Q.

From the perspective of phage gene expression and regulation, it is also possible that lyso‐lysis is simply a result of Int expression due to low levels of CII activity during the lytic process. The phage DNA integration is a highly regulated event, depending both on the integrase level as well as its competitor, the excisionase, Xis, which can alter the direction of recombination toward excision (Landy, 1989; Miller et al., 1981; Nash, 1981). During early infection*, int* is expressed from the pL promoter together with *xis* in the same transcript (Oppenheim et al., 2005), however, the *int* mRNA level is lower compared to *xis* (Fig. S3, first 5 min), due to retroregulation of *int* by the downstream DNA element *sib* (Schindler & Echols, 1981). DNA integration is therefore unlikely to happen during the very early infection period. Later, for cells committing to the lysogenic pathway, *int/xis* expression from pL will be shut down by CI, while the pI promoter is activated by CII, allowing only *int* to be expressed (Oppenheim et al., 2005), resulting in DNA integration. During lyso‐lysis, Int must also be expressed to reach sufficient levels for integration. It is possible that a low level of CII is present to promote Int expression from the pI promoter. If so, the level might be too low to either affect other phage DNA or activate pRE to commit to lysogeny, allowing lysis to proceed. In fact, the *int* mRNA level after λ*cII*_*68*_ infection is substantial (Fig. S3). Since λ*cII*_68_ infection leads to >99% of lytic cells, especially at a low APIs (Fig. 4E), it appears that lytic cells have significant CII protein. This relatively high level of *int* mRNA level is most likely due to the high copy number of phage DNA templates available in lytic cells, as phage DNA replicates to such an extent that not all DNA can be packaged to produce viable phages. This means that there is an excess of unused lambda DNAs in lytic cells, and integration might be a good strategy for cells to utilize the free lambda DNA as a backup, should lysis unexpectedly fail.

Lysogens were reported to have a high frequency of having multiple phage DNA integrated even at low API (Table S1), as reported previously (Freifelder & Kirschner, 1971; Freifelder & Levine, 1973) and kinetic studies on the DNA integration process suggest multiple phage DNAs can either integrate sequentially or all at once (Freifelder & Levine, 1973, 1975). Whether all the lambda DNAs inside the cell will be integrated into the genome or not and what happens to the rest of lambda DNA remain unknown. Here, our observation that some phage DNA integrated very late while the cells seemed to have entered the lysogenic pathway (Fig. 3F) suggest that some replicated unlabeled phage DNA may integrate early while the others, that is, the labeled DNA, diffuse throughout the cell until they are also integrated.

We observe that *attB* migrates to the cell pole in lytic cells, where phage DNA is more enriched as shown in Figure 3G. This movement pattern is very similar to the prophage integration in lysogenic cells (Tal et al., 2014). In our system, due to the limited number of lysogens obtained, the relatively low time resolution (5 min) and long preparation times (~10 min), most of integration happens within the first 20 min under the microscope. Therefore, we are unable to observe the detailed *attB* and phage DNA relative movements prior to integration in cells committed to lysogeny. However, for lytic cells, it is possible that the *attB* migration leads to more occurrence of colocalization of *attB* and lambda DNA, and thus, whenever Int is present, DNA integration can happen. This *attB* migration happens without the presence of Int, which leaves the driving force unknown. Further analysis reveals that in lytic cells the *attB* number does not increase significantly (Fig. S4C), suggesting that the lambda lytic development inhibits the host DNA replication to some extent, possibly due to the competition of limited resources by actively replicating phage DNA (Mallory et al., 1990). Moreover, the length extension of lytic cells is slower compared to lysogenic cells (Fig. S4B). Therefore, lack of cellular division, compromised host DNA replication and slower length extension might contribute together to the drastic shift of *attB* distribution after lytic development.

## Funding Information

This work was supported by the National Institutes of Health (R01GM107597) and TAMU‐NSFC grant from Texas A&M University (02‐230242). B.C. was supported by the National Science Foundation (NSF‐REU #DBI‐1358941). G.B. was supported by the National Institutes of Health (R01GM107597) and Laufer Center for Physical & Quantitative Biology. The funders had no role in study design, data collection and interpretation, or the decision to submit the work for publication.

## Conflict of Interest

None declared.
