# Supplementary material for: Lysis‐lysogeny coexistence: prophage integration during lytic development
Source: Microbiologyopen. 2016 Aug 17;6(1):e00395. doi: 10.1002/mbo3.395 (PMC5300877; doi:10.1002/mbo3.395)
Supplement: Supplementary file 5 [file MBO3-6-0-s005.docx]

**Supporting Information to:**

**Lysis-lysogeny Coexistence: Prophage Integration during Lytic Development**

Qiuyan Shao,^1,2^ Jimmy T. Trinh,^1,2^ Colby S. McIntosh,^1^ Brita Christenson,^3^ Gabor Balazsi,^4^

and Lanying Zeng1^,2^*

1Department of Biochemistry and Biophysics,

^2^Center for Phage Technology, Texas A&M University, College Station, TX 77843, USA

^3^Department of Biology and Biochemistry, University of Northwestern - St. Paul, St. Paul, MN 55113, USA

^4^Laufer Center for Physical & Quantitative Biology, Department of Biomedical Engineering, Stony Brook University, Stony Brook, New York 11794, USA

^*^For correspondence. E-mail: [lzeng@tamu.edu](mailto:lzeng@tamu.edu). Tel: 979-845-2961. Fax: 979-845-9274.

**Contents:**

Figure S1. The validation of the reporter system.

Figure S2. Lysogenization frequency of λ*int^-^-Kan* is much lower compared to λWT.

Figure S3. The *cII*, *int* and *xis* mRNA levels along time for cells infected by λ*cII_68_*, λWT and λ*cII_stable_*.

Figure S4. Division inhibition, compromised host DNA replication and length extension during the λWT-FP lytic development.

Table S1. Percentage of multiple prophage integration at different APIs.

Table S2. Bacterial strains, plasmids, phages, and primers used in this work.

Movie S1. A time-lapse movie showing a typical lysis event.

Movie S2. A time-lapse movie showing a lysogenic event with DNA integration.

Movie S3. A time-lapse movie showing a lyso-lysis event where DNA integration happens in a lytic cell.

Movie S4. A time-lapse movie showing a false lyso-lysis event where DNA integration seems to happen in a lytic cell infected by λ*int^-^*.

**Figure S1.** The validation of the reporter system.

A. The distribution of SeqA binding sites on bacteriophage lambda genome. Each bar represents the number of GATC of every 1000 bp along the lambda genome. The GATC sites are fairly evenly distributed across the genome.

B. The distribution of lysogenic cells with different fraction of time of ‘co-localization’ within 0.5 μm after integration. The percentage of time for a cell having Dis(λ-*attB*) < 0.5 μm (indicating co-localization by our standard) after integration is calculated and the percentage distribution for all lysogenic cells (N = 44) is shown. The distribution shows that the majority of the cells have at least 90% of the time showing co-localization, i.e., Dis(λ-*attB*) < 0.5 μm after integration, indicating that the criterion for co-localization is reasonable. A low fraction of cells show lower frequency of having Dis(λ-*attB*) < 0.5 μm, which could be due to multiple integration of lambda DNA or the lost tracking of target lambda DNA/*attB* that move out of focal plane. Error bars represent ± SEM.

C. The lysis time of λWT-FP and λ*int^-^* is similar to each other. λWT-FP: green circle, λ*int^-^*: blue circle, Gaussian fit for λWT-FP (green line) and λ*int^-^* (blue line). The dashed vertical lines mark the center of the Gaussian fit. λWT-FP and λ*int^-^* show a similar lysis time.

D. Fully methylation of lambda DNA by pZA32-*dam* in *dam^+^* host. Genomic DNA of λWT is extracted after induction from the host as indicated (*dam^+^* or *dam^-^*) with or without the corresponding plasmid, pZA32-*dam* or pGG503 as a positive control. Samples in the upper lane are digested with *MboI*, an enzyme specific for unmethylated DNA while the lower lane with *DpnI*, which digests methylated DNA. Without digestion, the lambda DNA runs as a clear band on the top of the gel (lane 1, 3, 5, 7, 9, 11), and *MboI* can fully digest the unmethylated DNA produced from *dam^-^* host, resulting in smears while *DpnI* can not (lane 2). When phages are induced from the *dam^+^* host, the lambda DNA is partially methylated, indicated by the incomplete digestion by *MboI* (lane 8). As a positive control, when the plasmid pGG503 was provided to *dam^+^* and/or *dam^-^* host, the lambda DNA is fully methylated and cannot be digested by *MboI* (lane 6, 12). With pZA32-*dam*, some of the lambda DNAs produced from the *dam^-^* host are still unmethylated, indicated by the noticeable smear from the top band (lane 4), however all lambda DNA obtained from the *dam^+^* host are fully methylated (lane 10). The results indicate that pZA32-*dam* plasmid provides enough Dam methylase in the *dam^+^* host background to produce fully methylated lambda DNA.

**Figure S2.** Lysogenization frequency of λ*int^-^-Kan* is much lower compared to λWT. The lysogenization frequency of λ*int^-^-Kan* (red right triangle) is at least 100 fold lower compared to λWT (blue right triangle), confirming that the lambda integrase is non-functional for λ*int^-^-Kan*. The lysogens obtained from λ*int^-^-Kan* infections are likely coming from the low level read-through of the amber codon.

**Figure S3.** The *cII*, *int* and *xis* mRNA levels along time for cells infected by λ*cII_68_*, λWT and λ*cII_stable_*. The mRNA level is normalized to that of reference gene *ihfB*. λ*cII_68_*: green, λWT: blue and λ*cII_stable_*: red.

A. The *cII* mRNA level for cells infected by different phages shows the same trend, where *cII* reaches a peak at around 6 min after infection and subsequently decreases, likely due to degradation by RNase and inhibition of transcription by CI or Cro. The λ*cII_stable_* infection shows a lower peak, which is most likely due to the earlier establishment of lysogenic and therefore an earlier peak that we didn’t detect due to the limited time resolution. At the time of taking the 6 min sample, the *cII* level for λ*cII_stable_* is most likely already at its decreasing phase.

B. The *int* mRNA level is low early after infection due to degradation by RNase, and subsequently increases, possibly due to the activation of transcription from pI promoter by CII. λ*cII_stable_* infection leads to earlier and higher level expression of *int* compared to λWT and λ*cII_68_*.

C. The *xis* mRNA level. Cells infected by different phages show the same trend of *xis* mRNA level after infection along time. The *xis* mRNA level increases and reaches a peak at around 6 min after infection, and subsequently drops due to degradation and inhibition of transcription from pL promoter by CI or Cro.

**Figure S4.** Division inhibition, compromised host DNA replication and length extension during the λWT-FP lytic development. Lytic: green, lysogenic: red, and uninfected: black. Error bars represent ± SEM.

A. The average cell length of lytic, lysogenic and uninfected cells along time. For lysogenic cells, due to the constant cellular division, the cell length remains almost constant like uninfected cells, while the lytic cells show increase along time due to the division inhibition.

B. Total cell length per lineage of lysogenic, lytic and uninfected cells along time. For each initial cells detected, the cell lineage is determined and at each time point the total length of this lineage of cells is calculated, i.e., for a specific uninfected cell, the sum of cell length of all its progeny at a certain time point is calculated. The average of the sum length of each group of cells is then calculated and shown. It is obvious that at the beginning of infection, the length extension of lytic cells is equivalent to that of lysogenic and uninfected cells, however, after about 60 minutes the length extension slows down significantly.

C. The total number of *attB* for each cell lineage for lytic, lysogenic and uninfected cells. The *attB* number increases in lysogenic and uninfected cells along time, while it remains the same in lytic cells, indicating inhibition of host DNA replication by phages in the lytic development.

D. The average *attB* number per unit of length. Lysogenic and uninfected cells manage to keep a roughly constant number of *attB* per unit of length during growth, however, this number drops in lytic cells, most likely due to lack of host DNA replication.

| API | % Multiple integration |
| --- | --- |
| 0.1 | 48 ± 7 |
| 1 | 55 ± 7 |
| 10 | 70 ± 8 |

**Table S1.** Percentage of multiple prophage integration at different APIs.

| Bacterial strains, plasmids, phages, and primers | | |
| --- | --- | --- |
| Strain Name | Relevant Genotype | Source/Reference |
| Bacterial strains | | |
| MG1655 | *sup^0^* | Lab collection |
| LE392 | *sup^E^, sup^F^* | Lab collection |
| LZ722 | *MG1655*, *dam^-^*, *seqA-yfp*, 200×*tetO-attB*, *Cm^R^*, *Gm^R^* | This work |
| LZ731 | LZ722[pFtsKi-*tetR-mCherry*] | This work |
| Phage strains | | |
| λWT | Fully methylated, λ *cI_857_ bor::Kan^R^* | Lab collection |
| λWT-FP | Fully methylated, gpD-mosaic, λ *D-mTurquoise2* *cI_857_ bor::Kan^R^* | This work |
| λ*int^-^* | Fully methylated, λ *int(AM) cI_857_* | Jeffery Gardner |
| λ*int^-^-Kan* | λ *int(AM)* *cI_857_ bor::Kan^R^* | This work |
| λ*cII_68_* | λ *cI_857_ cII_68_ bor::Cm^R^* | This work |
| λ*cII_stable_* | λ *cI_857_ cII_stable_ bor::Cm^R^* | This work |
| Plasmids | | |
| pFtsKi-*tetR-mCherry* | *tetR-mCherry* under constitutive promoter pFtsKi, *Amp^R^* | This work |
| pPLate-*D* | gpD under the control of λ late promoter, *Amp^R^* | This work |
| pZA32-*dam* | Dam under the control of PLlacO-1 promoter, *Cm^R^* | This work |
| pBR322-*D-mTurquoise2-E* | *D-mTurquoise2* fusion and part of downstream gene *E* | Lab collection |
| Primers | | |
| QS1 (attBup-forward) | 5'-GCCGACAACAAAGTCAGGTT | This work |
| QS2 (attBup-reverse) | 5'-AAAAGAAGCGCAGAATTTCG | This work |
| QS3 (attB-forward-2) | 5'-AGACGGGAAACTGAAAATGTG | This work |
| QS4 (attP-reverse) | 5'-CTGATAGTGACCTGTTCGTTGC | This work |
| QS5 (ihfB-forward) | 5'-ACCACGTACCGGACGTAATC | This work |
| QS6 (ihfB-reverse) | 5'-ATCGCGCAGTTCTTTACCAG | This work |
| QS7 (cII-forward) | 5'-GCAGATCAGCAGGTGGAAGA | This work |
| QS8 (cII-reverse) | 5'-AATCGAGCCATGTCGTCGTC | This work |
| QS9 (int-forward) | 5'-ATGCCCGAGAAGATGTTGAG | This work |
| QS10 (int-reverse) | 5'-GCACGAAAAGCATCAGGTCT | This work |
| QS11 (xis-forward) | 5'-GCCACCTGTTACTGGTCGAT | This work |
| QS12 (xis-reverse) | 5'-AACAGTTCGTCGATGGGTTC | This work |
| QS13 (cI-forward) | 5'-GGGATCATTGGGTACTGTGG | This work |
| QS14 (cI-reverse) | 5'-TTGGGGGTGATGAGTTTACC | This work |
| QS15 (FtsKi-forward-1) | 5'-GAATTCCGCCGGTGAGCCGGTGGTTGCC | This work |
| QS16 (FtsKi-reverse) | 5'-AAGCTTATTAGTCAAACGGCGGTGGGGCCAGCAC | This work |
| QS17 (FtsKi-forward-2) | 5'-CCCGGGGGCCCTTTCGTCTTCAAG | This work |
| QS18 (mCherry-reverse) | 5'-GCTAGCGGATCCTTACTTGTACAGCTCG | This work |
| QS19 (Dam-forward) | 5'-ATCACCTAGGCCTAGGGTTTCATCCGCTTCTCC | This work |
| QS20 (Dam-reverse) | 5'-TGGAGGTACCGGTACCAGTCAGCATGAAGAAAAATCG | This work |

**Table S2.** Bacterial strains, plasmids, phages, and primers used in this work.

**Movie Legends**

**Movie S1.** A time-lapse movie showing a typical lysis event. The frames shown are overlay images of phase-contrast, mCherry, YFP and CFP channels. Yellow and red dots represent phage DNA and *E. coli attB* respectively. The cell goes to the lytic pathway as indicated by the accumulation of mTurquoise2 (cyan) representing phage production, and cell lysis at 180 min. At 0 min, the phage DNA shows up as a very bright yellow focus which subsequently separates into two foci. DNA co-localization between lambda DNA and *attB* happens only occasionally, which might result from random collision or imaging artifact. The scale bar is 1 μm.

**Movie S2.** A time-lapse movie showing a lysogenic event with DNA integration. The frames shown are overlay images of phase-contrast, mCherry, YFP and CFP channels. Yellow and red dots represent phage DNA and *E. coli attB* respectively. The cell enters the lysogenic pathway, indicated by the co-localization of lambda DNA and *attB*, normal cell division and lack of phage progeny build up. At 0 min, there is one YFP focus showing up, which then separates into two. Starting at 20 min, there are three YFP foci present in the cell, which indicates that the cell is initially infected by two phages, and that those two phages either have both ejected their DNA at 0 min yet they stick together, or that one phage ejects later at 20 min. Nevertheless, DNA co-localization can be observed starting from 25 min. The lambda DNA and *E. coli attB* seem to separate in spaces occasionally after 25 min, which could be due to the diffusion of different segments of the chromosome or multiple integrations. The scale bar is 1 μm.

**Movie S3.** A time-lapse movie showing a lyso-lysis event where DNA integration happens in a lytic cell. The frames shown are overlay images of phase-contrast, mCherry, YFP and CFP channels. Yellow and red dots represent phage DNA and *E. coli attB* respectively. The cell is infected by one phage which shows as an YFP focus at 2 min. After 62 min, DNA co-localization between lambda DNA and *attB* happens, and it lasts until cell lysis at 132 min. The scale bar is 1 μm.

**Movie S4.** A time-lapse movie showing a false lyso-lysis event where DNA integration seems to happen in a lytic cell infected by λ*int^-^*. The frames shown are overlay images of phase-contrast, mCherry, YFP and CFP channels. Yellow and red dots represent phage DNA and *E. coli attB* respectively. The cell is infected by one phage which shows up as an YFP focus at 0 min. DNA co-localization happens starting from 90 min and lasts until cell lysis at 133 min. The scale bar is 1 μm.
